# Ein modulares Modell zur Qualitätssicherung im Medizin- und Ernährungsjournalismus

**DOI:** 10.1007/s00103-020-03254-0

**Published:** 2020-12-02

**Authors:** Marcus Anhäuser, Holger Wormer, Astrid Viciano, Wiebke Rögener

**Affiliations:** 1 Institut für Kommunikations- und Medienwissenschaft, Universität Leipzig, Nikolaistraße 27–29, 04109 Leipzig, Deutschland; 2 Institut für Journalistik, Lehrstuhl Wissenschaftsjournalismus, Technische Universität Dortmund, 44221 Dortmund, Deutschland

**Keywords:** Journalistische Qualitätsforschung, Medizinjournalismus, Ernährungsjournalismus, Wissenschaftsjournalismus, Gesundheitskompetenz, Quality research in journalism, Medical journalism, Nutritional journalism, Science journalism, Health literacy

## Abstract

**Hintergrund und Ziele:**

Die Qualität medizinjournalistischer Beiträge spielt bei informierten Entscheidungen von Patienten, von politischen, wirtschaftlichen und gesellschaftlichen Akteuren sowie für die allgemeine Gesundheitskompetenz (Health Literacy) eine zentrale Rolle. Daher erscheinen Qualitätsstandards notwendig, die wissenschaftliche *und* journalistische Prinzipien berücksichtigen, aber auch flexibel auf Besonderheiten spezieller gesundheitsrelevanter Themenfelder (Medizin, Ernährung, Umwelt) skalierbar sind.

**Methoden:**

Im Rahmen des Medien-Doktor-Projekts wurden, ausgehend von einem internationalen Katalog, Kriterien für guten Medizinjournalismus analysiert, auf Basis theoretischer Konzepte und praktischer Anwendbarkeit neu klassifiziert und ergänzt. Parallel wurde ein Kriterienkatalog für guten Ernährungsjournalismus abgeleitet.

**Ergebnisse:**

Es konnte ein Konsens über einen Kriterienkatalog erzielt werden, der in allgemeinjournalistische, allgemeinwissenschaftsjournalistische und spezifisch medizinjournalistische Aspekte modularisiert ist. Dieser wird hier erstmals in einem Fachbeitrag vorgestellt. Medizinjournalistische Qualitätskriterien ließen sich mit wenigen Ausnahmen gut auf Ernährungsthemen anpassen. Auf Basis der beiden Kataloge werden seitdem regelmäßig weitere Medienbeiträge bewertet.

**Diskussion:**

Die stärkere Modularisierung der Kriterienkataloge erleichtert deren Anwendbarkeit und eventuell auch Ausweitung auf weitere Fachdisziplinen sowie die Nutzung durch Ärzte ebenso wie Laien. Während sich der Medizinjournalismus stark an wissenschaftlichen Evidenzkriterien orientiert, sollte für den Ernährungsjournalismus weiter untersucht werden, welche Rolle Studien und Experten im Vergleich zu anekdotischer Evidenz spielen.

## Abstract (en)

**Background and objectives:**

The quality of medical articles in journalism has a central role in informed decision-making by patients as well as by political, economic and social players, but also to general “health literacy”. Therefore, quality standards that take into account basic scientific *and* journalistic principles, but are also scalable to specific health-related topics (such as medicine, nutrition and environment) are particularly relevant.

**Materials and methods:**

Starting from an internationally established catalogue in the framework of the “media doctor project”, criteria for good medical journalism were analysed, reclassified and completed on the basis of theoretical concepts and practical applicability. In parallel, existing criteria from medical journalism were transferred to an adapted criteria catalogue for reporting on nutrition.

**Results:**

A consensus was reached on a catalogue of criteria modularised into general journalistic, general science journalistic and specific medical journalistic aspects, which is published here for the first time in a scientific paper. With some rare exceptions, quality criteria for medical journalism could be well adapted to nutritional topics. Based on the two catalogues, further media contributions have been regularly evaluated.

**Conclusions:**

The advanced modularisation of the criteria catalogues facilitates their applicability and possibly their extension to other disciplines and for use by medical doctors as well as by laypersons. While medical journalism is strongly oriented towards the criteria of scientific evidence, the role of academic studies and experts versus anecdotal evidence should be further investigated for nutritional journalism.

## Hintergrund

Journalistische Massenmedien spielen nach wie vor eine große Rolle bei der Information über gesundheitsrelevante Themen. Die Motive von Nutzerinnen und Nutzern sind dabei vielfältig – und keineswegs nur auf patientenrelevante Informationen beschränkt. Die Suche nach einer gesünderen Lebensweise bzw. mehr Wohlbefinden gilt dabei als wichtiges [1], einigen Untersuchungen zufolge sogar als dominierendes Motiv, noch vor Behandlungsmöglichkeiten [2]. Folgerichtig orientiert sich Medizinjournalismus nicht allein an Betroffenen oder besonders gesundheitsbewussten Zielpublika oder an der Zielgruppe der Ärztinnen und Ärzte, sondern auch an einem lediglich latent medizininteressierten Publikum: „Im Idealfall wird ein qualitativ guter Beitrag in den allgemein-journalistischen Medien nicht nur von Rezipientinnen und Rezipienten wahrgenommen, *weil* es um ein medizinisches Thema geht, sondern sogar, *obwohl* es um ein medizinisches Thema geht …“ [3].

Für die Bewertung der Qualität von Medienberichten mit Gesundheitsbezug bedeutet dies, dass diese nicht *allein* nach wissenschaftlich-medizinischen Kriterien bewertet werden dürfen, sondern auch allgemeinjournalistische Kriterien zu berücksichtigen sind, die Attraktivität und Verständlichkeit der Beiträge für ein breites Publikum adressieren. Für den Ernährungsjournalismus sollte dies in noch größerem Maße gelten als für den Medizinjournalismus, da hier oft Motive des Wohlbefindens und der Prävention gegenüber konkreten Therapien dominieren dürften. Im Folgenden möchten wir daher zunächst auf a) etablierte allgemeinjournalistische Kriterien, b) etablierte medizinjournalistische Kriterien und c) auf mögliche Kriterien für den Ernährungsjournalismus eingehen.

Die journalistische Qualitätsforschung hat entlang von Befragungen auf Basis normativ-demokratietheoretischer Aufgabenzuweisungen aus marktorientierter oder systemtheoretischer Perspektive eine Reihe von Qualitätskriterien formuliert, über die unter Journalistinnen und Journalisten verschiedener Ressorts ein weitreichender Konsens erzielt werden kann [4–7]; eine tabellarische Übersicht liefern z. B. Beck et al. [8]. In Anlehnung an solche im Journalismus generell verbreiteten Standards wurden daraus speziell für den Medizinjournalismus beispielsweise folgende Kriterien abgeleitet [9]:

- *Vielfalt* – einerseits verstanden als Themenvielfalt von Vorsorge, Erkennung, Behandlung bis zur Erforschung von Krankheiten und damit verbundenen politischen, wirtschaftlichen und ethischen Fragen, andererseits verstanden als Themen‑, Quellen- und Meinungsvielfalt,
- *Vollständigkeit* – bezogen auf notwendige Informationen, inklusive wesentlicher Aspekte einer Krankheit und von Studien, aber auch bezogen auf Quellentransparenz,
- *Relevanz* – bezogen auf die Themenwahl, auch erzeugt durch Nutzwert und Aktualität sowie Konsultation zuverlässiger Experten,
- *Verständlichkeit –* etwa durch Vermeidung komplexer Formulierungen, gut gegliederte, prägnant gefasste und anregend gestaltete Texte (vgl. z. B. Hamburger Verständlichkeitsmodell),
- *Sachlichkeit* – im Sinne einer rationalen, nicht übermäßig emotionalen oder dramatisierenden Darstellung,
- *Unabhängigkeit* – im Sinne einer Prüfung von Neuigkeiten, ob es sich tatsächlich um wissenschaftliche Fortschritte handelt sowie kritischer Distanz zu Public Relations (PR), etwa der Pharmaindustrie.

Zu diesem primär aus dem allgemeinen Journalismus abgeleiteten Vorschlag weisen aus der Medizin heraus entwickelte Kriterien eine große Schnittmenge auf. Doch sind frühe Ansätze wie der „Index of scientific quality for health reports in the lay press“ [10] noch fast ausschließlich geprägt von wissenschaftlich-medizinischen Gesichtspunkten. Spätere Projekte zum Qualitätsmonitoring der Medizinberichterstattung („Media Doctor Australia“ oder „HealthNewsReview“ USA; [11–13]) integrieren bereits einige explizit im Journalismus hervorgehobene Qualitätselemente wie „Quellenvielfalt“ oder „mehr als eine Pressemitteilung“.

Das erste deutschsprachige am Lehrstuhl Wissenschaftsjournalismus der TU Dortmund 2010 zunächst vor allem mit Mitteln der Robert Bosch Stiftung begonnene Projekt zum Qualitätsmonitoring der Medizinberichterstattung „Medien-Doktor“ (www.medien-doktor.de) hat die in Australien, Kanada und den USA etablierten Kriterienkataloge aufgegriffen, diese aber um 3 allgemeinjournalistische Kriterien ergänzt (*Themenwahl* (Aktualität/Originalität/Relevanz), *Verständlichkeit/Vermittlung* und *Faktentreue/Richtigkeit* [14]). Deren prinzipielle Anwendbarkeit wurde auch für Medizinpressemitteilungen erprobt [15]. Gleichwohl wurde im Sinne der internationalen Vergleichbarkeit die übrige Struktur der Kataloge zunächst beibehalten. Ein kursorischer Vergleich der Bewertungen in den verschiedenen Ländern deutet an, dass für die evidenzbasierte Medizin (EbM) zentrale Angaben zu Nutzen und Risiken sowie zum Evidenzgrad („Belege“) medizinischer Interventionen zu den am seltensten „erfüllten“ Kriterien gehören (Tab. 1).

**Table 1:** 

Medien-Doktor (Deutschland)			HealthNewsReview (USA)		Media Doctor (Australien)	
	*Anteil (%) Kriterium „erfüllt“* *in n* *=* *310 Beiträgen (2016)*		*Anteil (%) Kriterium „erfüllt“* *in n* *=* *500 Beiträgen (2008)*		*Anteil (%) Kriterium „erfüllt“* *in n* *=* *1230 Beiträgen (2008)*	
1	Belege	25	Kosten	23	Risiken	18
2	Risiken	27	Nutzen	28	Kosten	36
3	Nutzen	30	Risiken	33	Nutzen	36
4	Kosten	36	Belege	35	Belege	37
5	Experten/Quellen	41	Alternativen	38	Experten/Quellen	39
6	Mehr als Pressemitteilung	60	Experten/Quellen	56	Alternativen	51
7	Alternativen	59	Mehr als Pressemitteilung	65	Verfügbarkeit	56
8	Verfügbarkeit	72	Kein Disease Mongering	70	Mehr als Pressemitteilung	70
9	Neuheit?	74	Verfügbarkeit	70	Neuheit?	70
10	Kein Disease Mongering	83	Neuheit?	85	Kein Disease Mongering	83

*Die Prozentangaben geben jeweils den Anteil von begutachteten Beiträgen an, in denen ein Kriterium auf der Basis von mindestens 2 Gutachten als „erfüllt“ bewertet wurde. Quellen: eigene Daten, *[12, 13]

Wenngleich sich das internationale Kriterienraster auch in Deutschland als gut anwendbar erwies, ergaben sich Einschränkungen. So sind viele Kriterien ausgerichtet auf journalistische Beiträge über *eine* Intervention zu *einem* Krankheitsbild. Daneben erwiesen sich einige Kriterien als nur bedingt sinnvoll für Beiträge zur Gesundheitsprävention (etwa „Kosten“, wenn es um Studien zu Effekten von sportlicher Aktivität oder bestimmten Ernährungsweisen ging). Ferner waren einige Kriterien mitunter zu komplex in der Anwendung, weil darin zu viele Teilaspekte zu berücksichtigen waren. Als unbefriedigend empfunden wurde aus journalismustheoretischer Sicht, dass das internationale Kriterienraster nicht trennscharf zwischen primär wissenschaftlich-medizinischen und universellen journalistischen Kriterien (Beispiel: „Mehr als Pressemitteilung?“) gegliedert war. Diese offensichtliche Inkonsistenz erschwerte gelegentlich die Erläuterung der Kriterien.

Hieraus resultierten folgende Forschungsfragen:

1. Wie lässt sich ein international erprobter und in Deutschland weiterentwickelter Kriterienkatalog zur Qualitätsbewertung medizinjournalistischer Beiträge so strukturieren, dass er stringenter anwendbar und journalismustheoretisch besser vermittelbar ist?
2. Wie muss ein Kriterienkatalog aussehen, mit denen Gutachterinnen und Gutachter die Qualität journalistischer Beiträge über *Ernährungs*themen mit Gesundheitsbezug bewerten können?

Ziel der hier vorgestellten Arbeit war es also, einen bereits existierenden Kriterienkatalog für den Themenbereich Medizin zu optimieren sowie für einen weiteren Berichterstattungsgegenstand (Ernährungswissenschaften) neu zu entwickeln und anzuwenden. Die jeweiligen Kataloge dienen zum einen als Hilfestellung für Journalisten, können aber zum anderen auch von Ärzten ebenso wie von Laien als Bewertungshilfe der Medienberichterstattung über entsprechende Themen herangezogen werden. Im Folgenden wird jeweils nacheinander das Vorgehen für den Themenkomplex „Medizin“ und „Ernährung“ dargestellt. Abschließendes Ziel war es, verwandte Kriterien zu ermitteln, diese jeweils in Modulen zusammenzufassen und in einer gemeinsamen Kriterienübersicht für die verschiedenen Themenfelder darzustellen.

## Methode

### Überarbeitung des medizinjournalistischen Kriterienkatalogs

Zur Überarbeitung des existierenden Katalogs wurden die in Tab. 1 dargestellten Kriterien zunächst daraufhin analysiert, inwieweit sie a) spezifisch für die Darstellung von medizinischen Interventionen sind, b) eher allgemeinwissenschaftliche Prinzipien beschreiben oder c) auch in gängigen Kriterienkatalogen der allgemeinen Journalismusforschung zu finden sind. Zudem wurden die im deutschen Medien-Doktor-Projekt enthaltenen Kriterien *Themenwahl, Verständlichkeit/Vermittlung* und (allgemeine) *Faktentreue/Richtigkeit* entlang der Literatur dahin gehend überprüft, ob diese in ihrer Strukturierung gängigen allgemeinjournalistischen Kriterien entsprechen.

**Figure 1:**
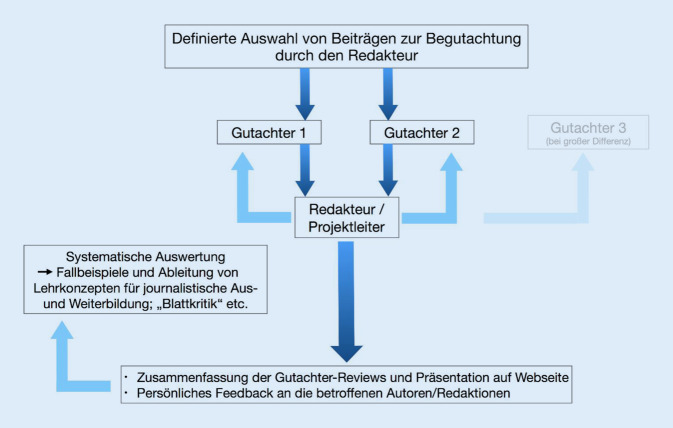

Im Rahmen weiterer Qualitätsbewertungen (Begutachtungen) im Rahmen des laufenden Medien-Doktor-Projekts wurde verstärkt darauf geachtet, inwieweit Schwierigkeiten bei der Anwendung bestimmter Kriterien auftraten. Dabei erfolgt die Begutachtung – wie seit Beginn des Gesamtprojekts – stets nach dem Vorbild eines Peer-Reviews auf der Basis von mindestens 2 Gutachtervoten (Abb. 1). Als Gutachter fungieren besonders ausgezeichnete Medizinjournalistinnen und -journalisten. Die Begutachtung durch echte „Peers“ verspricht breite Akzeptanz in der journalistischen Community.

Rückmeldungen aus den Begutachtungsprozessen wurden in mehreren Konsensfindungsrunden der Redaktionsleitungen für die 3 Teilprojekte „Medien-Doktor Umwelt“ (mit eigenem Kriterienkatalog), „Medien-Doktor Ernährung“ und „Medien-Doktor Medizin/Gesundheit“ diskutiert sowie retrospektiv mit Rückmeldungen aus der Bewertung der vorangegangenen *n* = 310 Gutachten (Zeitraum Nov. 2010 bis Nov. 2016) aus dem Medizinjournalismus verglichen. In einem letzten Schritt wurde ein überarbeiteter Kriterienkatalog [17] den aktuellen Gutachterinnen und Gutachtern vorgelegt und von diesen freigegeben. Das erste Gutachten auf Basis des neuen Katalogs wurde am 24.06.2020 öffentlich zur Diskussion gestellt [18]. Seitdem werden auf Basis des neu entwickelten Katalogs regelmäßig weitere Begutachtungen von Medienbeiträgen vorgenommen und auf der Webseite veröffentlicht.

### Entwicklung von Kriterien zur Bewertung ernährungsjournalistischer Beiträge mit Gesundheitsbezug

Die Entwicklung und Erprobung von Qualitätskriterien für den Ernährungsjournalismus orientiert sich am Entstehungsprozess eines Katalogs für die Umweltberichterstattung [19]. In einem ersten Schritt wurde dafür in einem Set von Ernährungs‑, Kommunikations- und sozialwissenschaftlichen Fachzeitschriften sowie Webseiten fachjournalistischer Verbände nach Kriterien oder Empfehlungen für den Ernährungsjournalismus gesucht (Liste kann angefordert werden).

Hierzu wurden folgende Suchbegriffe verwendet: „quality“, „criteria“ „food“, „diet“, „nutrition“, „journalis*“, „communication“, „evaluation“, „media“, „consumer information“, „public communication“, „media coverage“. Dabei finden sich vor allem Empfehlungen für die Kommunikation durch Wissenschaftler oder für die Öffentlichkeitsarbeit. Nur vereinzelt gibt es Hinweise auf Kriterien für den Ernährungsjournalismus.

So gibt die American Dietetic Association (ADA) Tipps für die Berichterstattung in Form eines Fragenkatalogs, wie z. B. „Wurde die Studie von einer glaubwürdigen Institution und einem qualifizierten Wissenschaftler durchgeführt?“ [20]. In einer Untersuchung zur Rolle der Medien in der Ernährungskommunikation wird empfohlen, mehrere Studien und Experten einzubeziehen und die Finanzierung zu berücksichtigen [21]. Dem Thema Interessenkonflikte in der Ernährungswissenschaft widmet McNutt einen ganzen Beitrag [22]. In einer Analyse der Berichterstattung über Kontaminationen in Lachsfarmen [23] raten die Autoren zu einer einordnenden Form bei der Berichterstattung von Gesundheitsrisiken durch Nahrungsmittel. Eine systematische Liste von Kriterien, mit der die Qualität eines ernährungsjournalistischen Beitrags bestimmt werden könnte, wurde nicht gefunden. Einige Einzelkriterien weisen jedoch Bezüge zu bereits im Medizinjournalismus verwendeten Kriterien auf, etwa:

- Expertenauswahl,
- Einordnung der Belege/Studien,
- sprachliche Darstellung/Vermittlung,
- (zweite) Quelle,
- Interessenkonflikte,
- Einordnung von Risiken.

Mangels eines Kriterienkatalogs aus der wissenschaftlichen Literatur wurde daher das vorliegende Kriterienraster für den Medizinjournalismus als Ausgangspunkt gewählt. In dem Sample mit *n* = 310 medizinjournalistischen Bewertungen wurden dazu vorhandene Gutachten zu Beiträgen mit Ernährungsbezug gesucht und retrospektiv daraufhin überprüft, inwieweit Schwierigkeiten bei der Anwendbarkeit medizinjournalistischer Kriterien dokumentiert worden waren.

Auf dieser Basis wurde die Nutzbarkeit medizinjournalistischer Kriterien für ernährungsjournalistische Beiträge mit Gutachtern aus der Praxis in mehreren Konsensfindungsrunden zwischen September 2019 und April 2020 diskutiert. Bei den Beteiligten handelt es sich um Journalistinnen und Journalisten, die intensiv über Ernährungsthemen berichten (teils in Büchern oder fachspezifischen Blogs oder innerhalb ihrer redaktionellen Tätigkeit für renommierte Medien) und die über verschiedene Wege (z. B. Journalistenpreise) identifiziert wurden. Insgesamt 11 ernährungsjournalistische Gutachter waren schließlich in den Prozess der Kriterienentwicklung eingebunden; 9 von ihnen sind derzeit (Stand November 2020) an den Begutachtungsverfahren beteiligt [24].

Um die überarbeiteten Qualitätskriterien auf ihre erste Anwendbarkeit zu testen, wurden 4 Runden mit Testgutachten durchgeführt. Hierzu wurden 3 aktuelle ernährungsjournalistische Artikel aus Onlinepublikumsmedien ausgewählt. Die Auswahl der Artikel erfolgte im Sinne einer stratifizierten Zufallsauswahl immer nach folgenden Parametern: frei zugänglich, über Google-News-Suche mit den Suchbegriffen „Ernährung“ oder „Lebensmittel“ auffindbar, aus einem journalistischen Publikumsmedium aus Deutschland (Leitmedien, überregionale oder regionale Nachrichtenportale, Onlineportale von Zeitschriften- und Lifestylemagazinen). Der Beitrag sollte ferner über Effekte eines Lebens- oder Nahrungsmittels, eine Ernährungsform, eine Diät oder eine Substanz berichten. Für die Testgutachten sollte *ein* Beitrag durch eine wissenschaftliche Studie veranlasst sein (Testgutachten 1), *ein* Beitrag einen nichtwissenschaftlichen Anlass haben (Testgutachten 2) und *ein* Artikel sollte einen Vortrag zusammenfassen oder allein auf Aussagen z. B. eines Ernährungsberaters und Buchautors beruhen (Testgutachten 3). Nachdem die ersten 3 Testbeiträge eher kritisch bewertet worden waren, wurde kontrastierend als vierter Beitrag ein bereits im o. g. Sample von *n* = 310 Beiträgen nach medizinjournalistischen Kriterien als sehr gut bewerteter Artikel ausgewählt ([25], Testgutachten 4). Damit sollten die Gutachterinnen und Gutachter die Gelegenheit bekommen, einen potenziell hochwertigen Beitrag zu bewerten, und die Kriterien weiter auf Plausibilität geprüft werden.

Auf Basis der Testgutachten und der Konsensfindungsrunden wurde ein Kriterienkatalog für ernährungsjournalistische Themen erarbeitet, der dann analog zur Neustrukturierung des Katalogs für die Medizinberichterstattung (s. oben) modularisiert wurde. Die finalen Kriterien wurden vor dem Start der regulären Begutachtung nochmals mit den ernährungsjournalistischen Gutachtern konsentiert. Alle Gutachten sind seit dem 23.06.2020 auf dem Monitoringportal [26] online zugänglich. Am jeweiligen Begutachtungstag werden aus der Trefferliste der Google-News-Suche immer jene Beiträge herausgesucht, die den o. g. Auswahlkriterien entsprechen, aus denen dann ein Beitrag zufällig ausgewählt und anonymisiert zur Begutachtung verschickt wird. Bewertungsgrundlage sind die konsentierten Kriterien, die jeweils zusammen mit kurzen Begründungen als „erfüllt“, „nicht erfüllt“ oder „nicht anwendbar“ bewertet werden.

## Ergebnisse

### Überarbeitung und Modularisierung des medizinjournalistischen Kriterienkatalogs

Als Ergebnis des Konsensfindungsprozesses wurden 6 Kriterien zur Beschreibung von *Nutzen, Risiken und Nebenwirkungen, Verfügbarkeit,* Darstellung von *Alternativen* und *Kosten* einer medizinischen Intervention sowie von Vermeidung eines *Disease Mongering* (Erfinden oder Übertreiben von Krankheiten) als speziell medizinjournalistische Kriterien beibehalten. Diese Kriterien waren in früheren Katalogen, Prinzipien der (evidenzbasierten) Medizin folgend, enthalten oder weisen eine direkte Patientenorientierung auf. Der Pressekodex des Deutschen Presserats formuliert spezielle Anforderungen für die Medizinberichterstattung, die in Bezug zur Darstellung von Nutzen, Risiken und der Verfügbarkeit stehen [27]: „Bei Berichten über medizinische Themen ist eine unangemessen sensationelle Darstellung zu vermeiden, die unbegründete Befürchtungen oder Hoffnungen beim Leser erwecken könnten. Forschungsergebnisse, die sich in einem frühen Stadium befinden, sollten nicht als (…) nahezu abgeschlossen dargestellt werden.“ Die Zuordnung von Krankheitsübertreibungen als Spezifikum der Medizinberichterstattung ist selbsterklärend [28].

Nicht beibehalten wurde das Kriterium *Neuheit*. Damit sollte bewertet werden, ob eine dargestellte Methode oder Arznei tatsächlich einen Fortschritt bedeutete oder es sich zum Beispiel lediglich um ein Nachahmerpräparat (Me-too-Präparat) handelt. Allerdings erwies sich die Anwendung des Kriteriums als schwierig; so wurde im journalistischen Verständnis „Neuheit“ irrigerweise oft mit Aktualität assoziiert. Zudem gehörte es zu den am häufigsten erfüllten Kriterien (Tab. 1). Im Konsensfindungsprozess wurde daher entschieden, dieses Kriterium in ein umfassenderes „Kontext“-Kriterium zu integrieren. Damit soll insgesamt geprüft werden, ob die im journalistischen Beitrag vorgestellten Inhalte in einen allgemeineren Kontext gestellt werden. Hierzu gehören insbesondere ethische Aspekte. Das neu entwickelte Kriterium soll sicherstellen, dass eine der ethisch-gesellschaftlichen Diskussion angemessene Darstellung erfolgt. Da der Anspruch der Kontextualisierung (der Beobachtung des „Interdependenzverhältnisses von Wissenschaft und Gesellschaft“) für die Wissenschaftsberichterstattung generell gefordert wird [29], wurde das Kriterium einer neuen Gruppe von für alle Formen der Wissenschaftsberichterstattung zentralen Kriterien zugeordnet.

In diese Gruppe wurden ferner die Kriterien „Belege“ und „Experten/Interessenkonflikte“ aufgenommen. Wie sich bei der Analyse ergab, stellen auch diese Kriterien keine auf Medizinjournalismus beschränkten Qualitätsmerkmale dar, sondern sollten – wenngleich im Detail anders operationalisiert – für die Wissenschaftsberichterstattung insgesamt betont werden. Das bisherige Kriterium „Experten/Interessenkonflikte“ wurde in 2 Einzelkriterien aufgeteilt, da in einem Beitrag zwar einerseits mehrere Experten zitiert werden können, andererseits aber ein Interessenkonflikt eines Experten womöglich nicht dargelegt wird. Während das Teilkriterium „Experte“ in einem solchen Fall als „erfüllt“ zu werten gewesen wäre, wäre das gleich gewichtete Teilkriterium „Interessenkonflikte“ indes „nicht erfüllt“. Das Kriterium (mehrere) „Experten/Quellen“ wurde daher um den Aspekt „Quellentransparenz“ ergänzt und der mit zunehmend drittmittelfinanzierter Forschung wichtige Aspekt „Interessenkonflikte“ als eigenständiges Kriterium für alle Bereiche der Wissenschaftsberichterstattung etabliert.

Das in bisherigen Katalogen medizinjournalistisch verortete Kriterium, wonach ein journalistischer Beitrag über eine Pressemitteilung hinausgehen muss, wurde den allgemeinjournalistischen Kriterien zugeordnet. So heißt es im Pressekodex [27], unabhängig vom Berichterstattungsgegenstand (Richtlinie 1.3): „Pressemitteilungen müssen als solche gekennzeichnet werden, wenn sie ohne Bearbeitung durch die Redaktion veröffentlicht werden.“

Auch die 2010 im deutschen Medien-Doktor-Katalog eingeführten allgemeinjournalistischen Kriterien „Themenwahl“ (unterteilt in Aktualität/Relevanz/Originalität), „Vermittlung“ und „Faktentreue“ wurden überprüft. So führen gängige Kataloge „Aktualität“ und „Relevanz“ als eigenständige Kriterien (vgl. Übersicht in Beck et al. [8]), während „Originalität“ nur vereinzelt erwähnt wird [30]. Gleichwohl wurde mit Blick auf die bisherigen Begutachtungsprozesse entschieden, es bei diesem 3‑teiligen Kriterium zu belassen – zumal einige Autoren Aktualität auch als einen Relevanz herstellenden Faktor betrachten. Zu einem anderen Ergebnis führte die Diskussion des Kriteriums „Vermittlung“, das künftig nach „Verständlichkeit“ und „Vermittlung“ (im Sinne von Attraktivität) differenziert ist. So kann ein Text gut verständlich, aber dramaturgisch nicht interessant zu lesen sein. Daher wurde im Einklang mit der allgemeinjournalistischen Qualitätsforschung eine Aufgliederung in 2 Kriterien vorgenommen: „Verständlichkeit“ und „Attraktivität“ der Darstellung. Das Kriterium der allgemeinen „Faktentreue“ wurde beibehalten.

**Figure 2:**
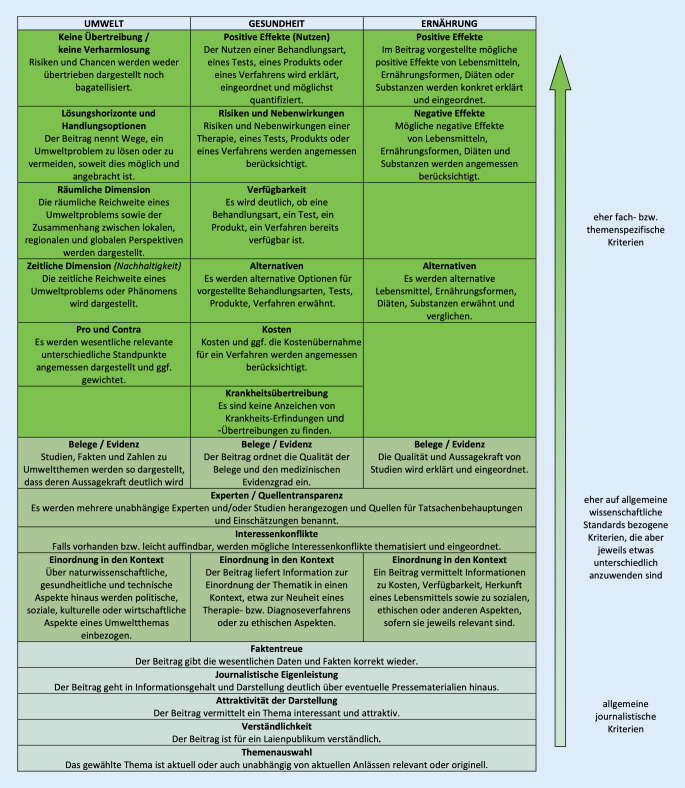

Der neu gefasste Katalog ist in Abb. 2 den Kriterien für Ernährungsjournalismus und Umweltjournalismus (auf den hier aus Platzgründen nicht eingegangen werden kann) gegenübergestellt (Langfassung zur Operationalisierung aller Kriterien: [17]).

### Katalog von Qualitätskriterien für den Ernährungsjournalismus

Die Auswertung von 13 identifizierten Gutachten zu Beiträgen mit Ernährungsbezug im Sample von *n* = 310 medizinjournalistischen Begutachtungen ergab, dass sich das Kriterium „Verfügbarkeit“ nur als bedingt geeignet erwiesen hatte: In 11 Fällen war es mit „erfüllt“, in 2 Fällen mit „nicht anwendbar“ gewertet worden – u. a. weil (anders als etwa bei Medikamenten) die Verfügbarkeit eines Lebensmittels oft als allgemein bekannt vorausgesetzt werden kann. Ähnlich verhält es sich beim Kriterium „Kosten“: Informationen dazu sind bei Ernährungsthemen nur von Bedeutung, wenn es zum Beispiel um Abnehmkonzepte wie Formula-Diäten oder Nahrungsergänzungsmittel geht, nicht aber bei Lebensmitteln des täglichen Bedarfs mit allgemein bekannten Preisen.

Dennoch können Aspekte von „Verfügbarkeit“ und „Kosten“ in Einzelfällen wichtige Informationen liefern, sodass sie nicht gestrichen, sondern in ein Kriterium „Kontext“ überführt wurden, in dem sie in begründeten Fällen berücksichtigt werden können. Ergänzt wurde dieses „Kontext“-Kriterium im Konsensfindungsprozess um weitere Aspekte wie die Herkunft und Produktionsweise eines Nahrungsmittels, z. B. von exotischen „Superfoods“, bio- oder gentechnisch erzeugten Nahrungsmitteln oder von Palmöl oder Avocados. In der Diskussion wiesen die Gutachterinnen und Gutachter darauf hin, dass ethische und soziale Aspekte von Bedeutung sein können, etwa das Körperbild junger Mädchen im Zusammenhang mit Diäten. Auch die Darstellung solcher Faktoren kann unter „Kontext“ bewertet werden.

Ein Aspekt ernährungswissenschaftlicher Forschung, der – neben der Frage nach ihrer hinreichenden Evidenz generell [31] – zuletzt an Bedeutung gewonnen hat, ist die Frage nach dem Einfluss großer Lebensmittelkonzerne und nach Interessenkonflikten von Experten [32–34]. Gutachterinnen und Gutachter wiesen in den Diskussionen zudem auf eine Problematik hin, die sowohl Aspekte der Interessenkonflikte als auch der Evidenz betreffen. Während im Medizinjournalismus meist auf akademisch ausgebildete Experten zurückgegriffen wird, finden sich in Berichten zu Ernährungsthemen häufig Vertreter aus der Ernährungsberatung oder Heilpraktiker ohne akademische Vorbildung. Diese können kompetente Experten sein, doch stellt sich die Frage, ob die Evidenzbasierung ihrer Aussagen äquivalent ist mit akademischer Expertise. Diesen Umständen tragen die schon bei der Neuordnung der medizinjournalistischen Kriterien eingeführten eigenständigen Kriterien zu „Interessenkonflikten“ und „Experten/Quellentransparenz“ Rechnung.

Die zentralen Kriterien im Medizinjournalismus „Darstellung des Nutzens“ sowie „Risiken und Nebenwirkungen“ wurden für den Ernährungsjournalismus allgemeiner beschrieben („positive Effekte“ und „negative Effekte“). Die Kriterien umfassen auch weniger gesundheitsrelevante Wirkungen, wie einen Gewichtsverlust aus ästhetischen Gründen, die Verdaulichkeit oder den erfrischenden Charakter einer Zutat. Das Kriterium „Disease Mongering“ wurde aufgelöst, da die übertriebene Darstellung von Erkrankungen bei Ernährungsthemen nicht den Stellenwert hat wie im Medizinjournalismus. In gravierenden Einzelfällen kann es unter „Faktentreue“ mitberücksichtigt werden.

In den 4 Testgutachten zeigte sich zum einen, dass die Gutachter die Qualität der Beiträge weitgehend übereinstimmend bewerteten. Der zuvor schon einmal von Medizinjournalisten begutachtete Beitrag wurde erneut positiv bewertet. Gleichwohl befindet sich der neu definierte Kriterienkatalog erst seit Frühjahr 2020 in der regulären Erprobungsphase, sodass evtl. die Notwendigkeit weiterer Anpassungen sichtbar werden könnte. Einen Überblick über alle Kriterien findet sich in Abb. 2, eine Langfassung zur Operationalisierung aller Kriterien unter [35].

## Diskussion

In diesem Beitrag wird ein modulares Bewertungssystem von Qualitätskriterien für den Medizin- und Ernährungsjournalismus beschrieben. Für den Medizinjournalismus wurde dargelegt, wie sich theorie- und anwendungspraktisch geleitet ein seit 2010 bestehender Kriterienkatalog in ein modulares System aus allgemeinjournalistischen, allgemeinwissenschaftsjournalistischen und spezifisch medizinjournalistischen Kriterien überführen lässt. Der überarbeitete, hier erstmals in einem Fachbeitrag vorgestellte und jetzt aus 15 Kriterien bestehende Katalog wurde durch medizinjournalistische Gutachterinnen und Gutachter positiv aufgenommen, wobei sich in der praktischen Anwendung auf weitere medizinjournalistische Beiträge zeigen muss, ob die Überarbeitung die erwünschten Verbesserungen bewirkt.

Für Beiträge aus dem Ernährungsjournalismus wurde in Anlehnung an medizinjournalistische Kriterien ein eigenes Kriterienset entwickelt. Auf Grundlage der Literaturauswertung und mehrerer Konsentierungsrunden resultierte ein Katalog aus 12 Kriterien, der dem medizinjournalistischen Katalog entsprechend modular strukturiert ist. Dabei wurden einige Kriterien aus dem Medizinjournalismus in einem „Kontext“-Kriterium vereint und ergänzt. 4 erste unveröffentlichte Testgutachten und mehrere auf der Projektwebseite veröffentlichte Gutachten lassen erste Hypothesen zu. So deutet sich ein seltenerer Verweis auf wissenschaftliche Studien an, um Aussagen zu Effekten von Lebensmitteln, Ernährungsformen, Diäten und Substanzen zu belegen. Des Weiteren ergibt sich aus den Gutachterdiskussionen die Hypothese, dass viele zitierte Experten aus dem nichtakademischen Bereich stammen, etwa Ernährungsberater oder Heilpraktiker. Dass der Aspekt „Interessenkonflikte“ verstärkt zu beachten sein könnte, illustriert exemplarisch ein Beitrag, in dem behauptet wird, dass Pasta kein Dickmacher ist, der aber auf einer Studie basiert, die von einem Nudelhersteller mitfinanziert wurde. Weitere Begutachtungen müssen zeigen, ob dies Hinweise auf Muster im Ernährungsjournalismus sind und dort womöglich quellenärmer berichtet wird als im Medizinjournalismus.

Perspektivisch bieten die modularisierten Kataloge gute Möglichkeiten, solche Fragen nun auf Basis mehrerer einheitlicher Einzelkriterien zu beantworten. Gleichwohl sei – neben dem Hinweis auf die erst angelaufene praktische Erprobungsphase – auch auf weitere Limitationen des dargestellten Vorgehens verwiesen: So ist die Stichprobenwahl beim „Medien-Doktor Ernährung“ aus frei verfügbaren Artikeln über die Google-News-Suche nicht repräsentativ für die deutsche Medienlandschaft; man könnte z. B. annehmen, dass hinter Bezahlschranken qualitativ hochwertigere Beiträge auffindbar wären. Da frei verfügbare Beiträge indes eine größere Reichweite haben, üben diese bereits großen Einfluss auf die Meinungsbildung aus. Generell ist einzuschränken, dass die hier vorgestellten Kriterien nur auf eher nachrichtliche Beiträge über Medizin- und Ernährungsthemen und somit nur auf einen (wenngleich erheblichen) Teil der Berichterstattung anwendbar sind. Zudem wird den (noch dazu in ihrer Zahl beschränkten) Gutachterinnen und Gutachtern trotz definierter Kriterien immer ein gewisser Ermessensspielraum bei der Bewertung zugestanden – wobei Letzteres auch bei einem wissenschaftlichen Peer-Review-Verfahren bewusst in Kauf genommen wird.

Perspektivisch erleichtert es die modulare Aufteilung der Kriterien, das Kriterienraster durch geringfügige Anpassungen auf weitere Themenfelder zu übertragen – etwa indem lediglich die medizinspezifischen Kriterien durch spezielle Kriterien zur Berichterstattung über wirtschafts- oder sozialwissenschaftliche Studien ausgetauscht werden, während die anderen Kriterien beibehalten werden können. Die neue Modulstruktur des Medien-Doktor-Projekts erleichtert also eine Anwendung des Bewertungsverfahrens in anderen Fachressorts, was gerade in Zeiten der Desinformation [36] von hoher Relevanz ist, um die Qualität der Berichterstattung mit Wissenschaftsbezug zu sichern. Darüber hinaus können solche klar strukturierten Kriterienkataloge aber auch Nutzerinnen und Nutzern, von der Ärzteschaft bis hin zu Laien, helfen, sorgfältig recherchierte von interessengeleiteten Beiträgen oder gar von Fake News zu unterscheiden. Die hier vorgestellten Kriterienkataloge sind dabei nur eine von mehreren Möglichkeiten, Qualitätsmaßstäbe zu operationalisieren, die perspektivisch (z. B. auch für neuere digitale Formate) ständig weiterzuentwickeln sind. Ihr unmittelbarer Wert besteht jedoch auch darin, dass sie von Fall zu Fall Impulse zur individuellen Reflexion über die Zuverlässigkeit von medial vermittelten Informationen geben.

## References

[1] Link E, Baumann E (2020). Nutzung von Gesundheitsinformationen im Internet: personenbezogene und motivationale Einflussfaktoren. Bundesgesundheitsblatt.

[2] Fromm B, Baumann E, Lampert C (2011). Gesundheitskommunikation und Medien. Ein Lehrbuch.

[3] Wormer H, Hurrelmann K, Baumann E (2014). Medizin- und Gesundheitsjournalismus. Handbuch Gesundheitskommunikation.

[4] Weber B, Rager G, Rager G, Haase H, Weber B (1994). Zeile für Zeile Qualität. Was Journalisten über Qualität in der Zeitung denken. Zeile für Zeile – Qualität in der Zeitung.

[5] McQuail D (1992). Media Perfomance. Mass communication and the public interest.

[6] Arnold K (2009). Qualitätsjournalismus. Die Zeitung und ihr Publikum.

[7] Arnold K (2008). Qualität im Journalismus – ein integratives Konzept. Publizistik.

[8] Beck K, Reineck D, Schubert C (2010) Journalistische Qualität in der Wirtschaftskrise. Berlin, S 24. https://www.polsoz.fu-berlin.de/kommwiss/arbeitsstellen/kommunikationspolitik/media/Studie_Journalistische_Qualitaet_03_2010.pdf. Zugegriffen: 30. Juni 2020

[9] Lilienthal V, Reineck D, Schnedler T (2014). Qualität im Gesundheitsjournalismus. Einleitung.

[10] Oxman AD, Guyatt GH, Cook DJ, Jaeschke R, Heddle N, Keller J (1993). An index of scientific quality for health reports in the lay press. J Clin Epidem.

[11] Moynihan R, Bero L, Ross-Degnan D (2000). Coverage by the news media of the benefits and risks of medications. New Eng J Med.

[12] Wilson A, Bonevski B, Jones A, Henry D (2009). Media reporting of health interventions: signs of improvement, but major problems persist. Plos One.

[13] Schwitzer G (2008). How do US journalists cover treatments, tests, products, and procedures? An evaluation of 500 stories. PLoS Med.

[14] Wormer H, Gigerenzer G, Gray JAM (2011). Improving health care journalism. Better doctors, better patients, better decisions: Envisioning health care 2020.

[15] Serong J, Anhäuser M, Wormer H (2015). Ein methodischer Ansatz zur Bewertung der Informationsqualität medizinisch-wissenschaftlichen Wissens auf dem Transferweg zwischen Fachpublikation und Massenmedien. Z Evid Fortbild Qual Gesundhwes.

[16] Wormer H, Anhäuser M, Lilienthal V (2013). „Gute Besserung! – und wie man diese erreichen könnte. Erfahrungen aus drei Jahren Qualitätsmonitoring Medizinjournalismus auf medien-doktor.de und Konsequenzen für die journalistische Praxis, Ausbildung sowie Wissenschafts-PR. Qualität im Medizinjournalismus.

[17] http://www.medien-doktor.de/gesundheit/bewertungen/die-kriterien/. Zugegriffen: 9. Nov. 2020

[18] http://www.medien-doktor.de/gesundheit/2020/06/erste-ergebnisse-entzuendungshemmer-hilft-bei-schwerem-covid-19/. Zugegriffen: 9. Nov. 2020

[19] Rögener W, Wormer H (2015). Defining criteria for good environmental journalism and testing their applicability: an environmental news review as a first step to more evidence based environmental science reporting. Public Underst Sci.

[20] Ayoob KT, Duyff RL, Quagliani D (2002). Position of the American Dietetic Association: food and nutrition misinformation. J Am Diet Assoc.

[21] Fernández-Celemín L, Jung A (2006). What should be the role of the media in nutrition communication?. Br J Nutr.

[22] McNutt K (1999). Conflict of interest. J Am Diet Assoc.

[23] Amberg SM, Hall TE (2010). Precision and rhetoric in media reporting about contamination in farmed salmon. Sci Commun.

[24] http://www.medien-doktor.de/ernaehrung/uber-uns/wer-sind-die-gutachter. Zugegriffen: 9. Nov. 2020

[25] medizin-aufs-brot. http://www.medien-doktor.de/gesundheit/2012/10/. Zugegriffen: 9. Nov. 2020

[26] http://www.medien-doktor.de/ernaehrung. Zugegriffen: 9. Nov. 2020

[27] Deutscher Presserat (2017) Publizistische Grundsätze (Pressekodex) Richtlinien für die publizistische Arbeit nach den Empfehlungen des Deutschen Presserats, Berlin. https://www.presserat.de/files/presserat/dokumente/download/Pressekodex2017light_web.pdf. Zugegriffen: 30. Juni 2020

[28] Moynihan R, Henry D (2006). The fight against disease mongering: generating knowledge for action. PLoS Med.

[29] Kohring M, Kienzlen G, Lublinski J, Stollorz V (2007). Vertrauen statt Wissen – Qualität im Wissenschaftsjournalismus. Fakt, Fiktion, Fälschung: Trends im Wissenschaftsjournalismus.

[30] Ruß-Mohl S (1992). „Am eigenen Schopfe …“: Qualitätssicherung im Journalismus – Grundfragen, Ansätze, Näherungsversuche. Publizistik.

[31] Ioannidis JPA (2018). The challenge of reforming nutritional epidemiologic research. JAMA.

[32] Soares MJ, Müller MJ, Boeing H (2019). Conflict of interest in nutrition research: an editorial perspective. Eur J Clin Nutr.

[33] Chartres N, Fabbri A, Bero LA (2016). Association of industry sponsorship with outcomes of nutrition studies. JAMA Intern Med.

[34] Ioannidis JPA, Trepanowski JF (2017). Disclosures in nutrition research: why it is different. JAMA.

[35] http://www.medien-doktor.de/ernaehrung/bewertungen/die-kriterien/. Zugegriffen: 9. Nov. 2020

[36] Nguyen A, Catalan-Matamoros D (2020). Digital mis/disinformation and public engagment with health and science controversies: fresh perspectives from Covid-19. Media Commun.

[37] Reinhart M (2012). Soziologie und Epistemologie des Peer Reviewy.

